# Severe hematuria after transurethral electrocoagulation in a patient with an arteriovesical fistula

**DOI:** 10.1186/1471-2490-13-68

**Published:** 2013-12-01

**Authors:** Xiangyi Zheng, Yiwei Lin, Bin Chen, Xianyong Zhou, Xiaofeng Zhou, Yuehong Shen, Liping Xie

**Affiliations:** 1 Department of Urology, The First Affiliated Hospital, Zhejiang University School of Medicine, 79# Qingchun Road, Hangzhou 310003, Zhejiang Province, China; 2 Department of Radiology, The First Affiliated Hospital, Zhejiang University School of Medicine, Hangzhou 310003, China; 3 Department of Urology, China-Japan Friendship Hospital, Beijing 100029, China

**Keywords:** Hematuria, Arteriovesical fistula, Vesical pseudoaneurysm

## Abstract

**Background:**

Arteriovesical fistulas are extremely rare. Only eleven cases were previously reported in the literature. They can occur iatrogenically, traumatically or spontaneously.

**Case presentation:**

We report an unusual case of a 62-year-old woman with arteriovesical fistula that developed fatal hematuria after transurethral electrocoagulation. Computed tomography (CT) and selective angiography revealed a pseudoaneurysm of the right superior vesical artery with arteriovesical fistula formation, which was managed by transarterial embolization.

**Conclusions:**

Contrast enhanced CT or CT angiography should be performed when a pulsatile hemorrhage is revealed during cystoscopy. Therapeutic vesical arterial embolization should be considered as a safe and effective procedure for arteriovesical fistulas. Transurethral electrocoagulation may cause severe hematuria for pulsatile bladder bleeding in patients with pelvic vascular malformation.

## Background

Arteriovesical fistulas are extremely rare. To our best knowledge, only eleven cases were previously reported in the literature. They can occur iatrogenically, traumatically or spontaneously [1]. We report an unusual case of a 62-year-old female with arteriovesical fistula formed spontaneously that developed fatal hematuria after transurethral electrocoagulation.

## Case presentation

**Figure 1:**
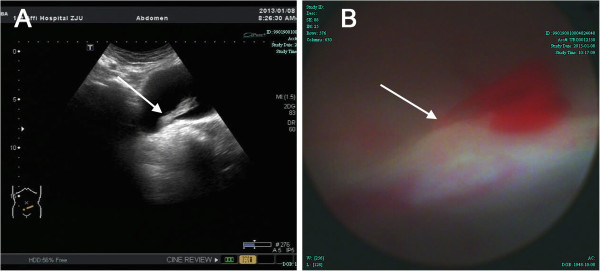
**Ultrasonography and cystoscopic finding of a woman with an 8-day history of painless gross hematuria. (A)** Ultrasonography revealed a long strip, 4.2x1.2 cm sized, hypoechogenic mass (*arrow*) floating in the bladder, its pedicle located at the bladder base. **(B)** Cystoscopic examination revealed a pulsatile hemorrhage site (*arrow*) from the right bladder base.

**Figure 2:**
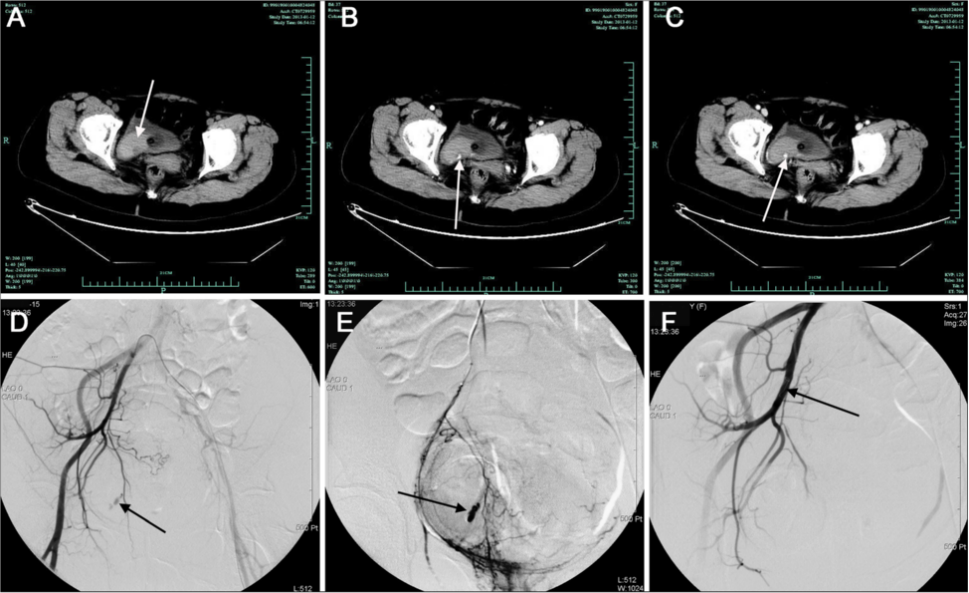
**Pelvic computed tomography and angiography showed the source of the bleeding managed by transarterial embolization. (A)** Noncontrast pelvic computed tomography (CT) revealed a blood clot *(arrow)* in the bladder; Contrast pelvic CT demonstrating the contrast blush suggestive of a pseudoaneurysm (arrow) **(B)** arterial phase CT image; **(C)** parenchymal phase CT image; **(D, E)** The pelvic angiography showing a pseudoaneurysm (arrow) of the superior vesical artery on the right side which was the source of the bleeding. **(F)** A successful endoluminal occlusion of the anterior trunk of the right internal iliac artery with a coil *(arrow).*

A 62-year-old woman presented to our outpatient clinic with an 8-day history of painless gross hematuria. She had seen local general physician a few days before this presentation and was prescribed antibiotic for a presumed urinary tract infection, which helpless. She has no significant past medical history and physical examination was unremarkable. Ultrasonography revealed normal upper urinary tracts and a long strip, 4.2 cm × 1.2 cm sized, hypoechogenic mass floating in the bladder, with its pedicle located at the bladder base (Figure 1A). Cystoscopic examination revealed blood clot and a pulsatile hemorrhage from the right bladder base (Figure 1B). She went back to local hospital and transurethral electrocoagulation was performed as initial treatment. The hematuria resolved immediately. Therefore, the Foley catheter were removed and the patient was discharged at 2 days post the first operation. One day after discharge, the patient represented to our emergency department with severe hematuria. The patient appeared to be hemodynamically stable; however, the bloods tests revealed a life-threatening drop in her hemoglobin levels from 12.3 to 6.4 g/dL (normal range 11.3-15.1 g/dL). The blood fibrinogen level was grossly abnormal at 0.4 g/L (normal range 2.0-4.0 g/L). Supportive transfusion was provided. A pelvic CT scan was implemented as a further investigation, which surprisingly showed dilated pri-vesical artery on the right-side, a blood clot in the bladder; and the contrast blush suggestive of a pseudoaneurysm (Figure 2A-C). Therefore, angiography with a retrograde catheterization from left common femoral artery was taken, which confirmed the right superior vesical arterial pseudoaneurysm as the source of bleeding (Figure 2D,E). A successful endoluminal occlusion of the anterior trunk of the right internal iliac artery embolization with coils was performed (Figure 2F). Urine gradually cleared during the next 2 days. Seven days later, she claimed only mild vesical pain which could be resolved by oral intake of solifenacin. Therefore, the Foley catheter was removed and the patient was discharged at 10 days after second operation.

**Figure 3:**
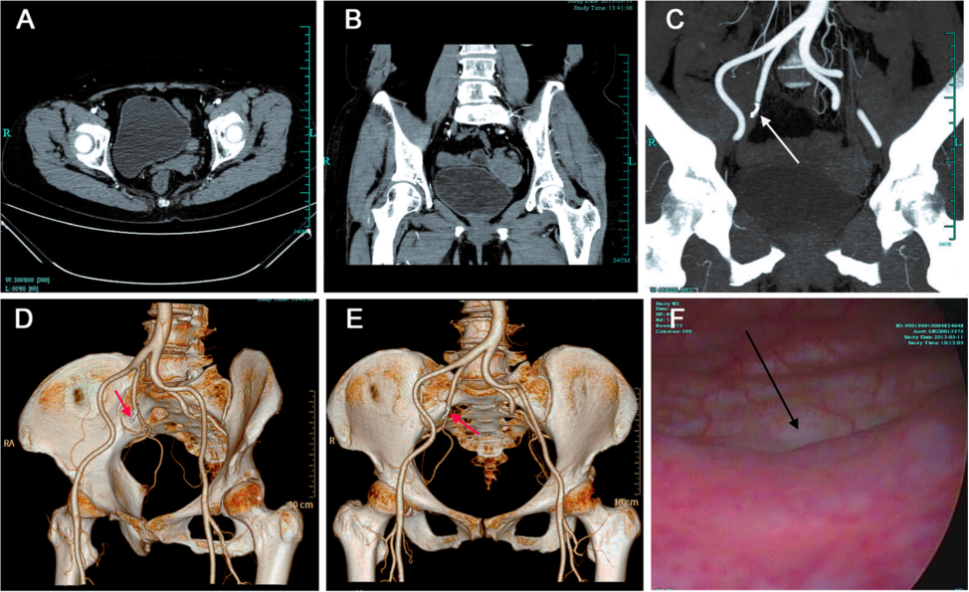
**A fellow-up pelvic computed tomography angiography (CTA) 2 months postoperatively showed completely occlusion of superior vesical artery with a coil *(arrow)*. (A)** arterial phase CT image; **(B)** image of coronal CT multiplanar reconstruction. **(C)** image of coronal CTA multiplanar reconstruction; **(D,E)** images of three-dimensional CTA. **(F)** Cystoscopic examination 2 months later revealed a healing mucosa after electrocoagulation *(arrow)* located at the right bladder base adjacent to interureteric fold.

During a follow up period of 4 months, CT angiography (CTA) revealed complete occlusion of superior vesical artery, and a duplex cystoscopy showed nomoral bladder (Figure 3). The patient is currently asymptomatic.

## Discussion

Hematuria due to arteriovesical fistula is an extremely rare clinical entity and we identified only a few cases in the literature. Arteriovesical fistulas can occur iatrogenic injury [2-6], secondary to trauma [1,7-9] and spontaneously [10,11]. Rous et al reported the first case of a ruptured posttraumatic pseudoaneurysm of the external iliac artery 1 week after a gunshot wound to the lateral aspect of the bladder [7]. Arteriovesical fistulas are mostly associated with previous pelvic surgeries [2-4], radiotherapy [5] and urologic interventions [6] in patients with vascular disease. There are only two cases where an arteriovesical fistula formed spontaneously due to a ruptured iliac artery aneurysm with hematuria as initial presentation [10,11].

A pseudoaneurysm, also known as a false aneurysm, is a leakage of arterial blood from an artery into the surrounding tissue with a persistent communication between the originating artery and the resultant adjacent cavity. The development of pseudoaneurysm of a vesical artery is extremely rare, and Pontin et al reported one similar case due to radiation therapy [5]. In this unique case presented, arteriovesical fistula was not recognized at first, and developed fatal hematuria after transurethral electrocoagulation for vesical bleeding. Further CT and selective angiography revealed a pseudoaneurysm of the right superior vesical artery with arteriovesical fistula formation. We postulated that a pulsatile bladder hemorrhage suggested a vascular malformation, maybe owing to rupture of a submucosal branch of vesical artery, and transurethral electrocoagulation causes damage to an arterial wall, which was responsible for the formation of the vesical arterial pseudoaneurysm. Rupture of a pseudoaneurysm is potentially a fatal event, particularly pseudoaneurysms that occur in large visceral arteries [12].

Establishing a precise diagnosis of superior vesical arterial pseudoaneurysm is often difficult since it used to be of rare occurrence. Still, it is important to be aware that vascular abnormality, such as aneurysm, fistula or malformation, could be a possible diagnosis for hematuria, especially when the bladder hemorrhage refractory to bladder irrigation, intra-vesical instillation or endo-urological intervention. Or characteristic pulsative bleeding was presented in cystoscopy inspection. Contrast enhanced CT or CTA would be a useful tool to identify the bleeding source.

There is no agreement in the literature on a specific course of treatment for arteriovesical fistula. Management options for the patients include embolization, open repair and aneurysm resection [9]. Selective and superselective embolization is the preferred treatment for patients with vascular pseudoaneurysms [12]. It is a safe and effective method, especially for those who are poor surgical candidates. Percutaneous vesical arterial embolization was first described in 1980 by Kobayashi et al [13]. In the present case, right internal iliac artery embolization successfully achieved an immediate hemorrhage control and no major post-embolization complications were noted, which indicated that vesical arterial embolization would be well-tolerated.

## Conclusions

In summary, we report a case of arteriovesical fistula formed spontaneously that developed fatal hematuria after transurethral electrocoagulation. Awareness of this as a possible cause of hematuria can assist in immediate diagnosis and appropriate treatment. Contrast enhanced CT or CT angiography should be performed when a pulsatile hemorrhage is revealed during cystoscopy. Therapeutic vesical arterial embolization should be considered as a safe and effective procedure for bleeding control. Transurethral electrocoagulation may cause severe hematuria for pulsatile bladder bleeding in patients with pelvic vascular malformation.

## Consent

Written informed consent was obtained from the patient for publication of this Case report and any accompanying images. A copy of the written consent is available for review by the Editor of this journal.

## Abbreviations

CT: Computed tomography; CTA: Computed tomography angiography.

## Competing interests

The authors declare that they have no competing interests.

## Authors’ contributions

XYZ, YWL drafted the manuscript. BC, XYZ, XFZ and LPX also assisted with manuscript preparation. YHS revised the manuscript. All authors have read and approved the final manuscript.

## Pre-publication history

The pre-publication history for this paper can be accessed here:

http://www.biomedcentral.com/1471-2490/13/68/prepub
